# Importance of Engaging Partners in Digital Postpartum Depression Prevention: Qualitative Study

**DOI:** 10.2196/71764

**Published:** 2025-12-08

**Authors:** Adam Korrick Lewkowitz, Liana Lum, Katrina Ursino, Melissa Guillen, Gabriela Garcia, Sarai K Sales, Saloni Taneja, Crystal F Ware, William A Grobman, Caron Zlotnick, Kate M Guthrie, Emily S Miller

**Affiliations:** 1 Department of Obstetrics & Gynecology Warren Alpert School of Medicine Brown University Providence, RI United States; 2 Center for Digital Health School of Public Health Brown University Providence, RI United States; 3 Warren Alpert School of Medicine Brown University Providence, RI United States; 4 Center for Behavioral and Preventive Medicine Brown University Health Providence, RI United States; 5 Department of Epidemiology School of Public Health Brown University Providence, RI United States; 6 Division of Biology and Medicine Brown University Providence, RI United States; 7 Department of Psychiatry and Human Behavior Warren Alpert School of Medicine Brown University Providence, RI United States; 8 Department of Health Services Policy & Practice School of Public Health Brown University Providence, RI United States

**Keywords:** digital perinatal mental health, postpartum depression prevention, nonbirthing partner, qualitative research

## Abstract

Participants in qualitative interviews designed to optimize the adaptation of a maternal postpartum depression intervention into a novel smartphone app noted that the app could be more useful if were delivered simultaneously to both parents; this calls for additional research on the feasibility and effectiveness of digital dyadic or stand-alone partner interventions to prevent postpartum depression.

## Introduction

Approximately 15% of women in the United States develop postpartum depression (PPD) [1]. The United States Preventive Services Task Force (USPSTF) recommends that women at risk for PPD participate in a program such as Mothers and Babies (MB), an in-person cognitive behavioral therapy (CBT)–based curriculum that prevents PPD [2]. Because smartphone app–provided CBT can reduce PPD symptoms and expand the reach of CBT programs [3], we used individual in-depth interviews with perinatal women to develop a novel app, MBapp, that adapts MB to a digital format [4].

During the interviews that guided app development, study participants noted that MBapp would be useful for their partner or would benefit their relationship with their partner. To explore MBapp’s potential utility as a dyadic intervention, we conducted a qualitative analysis of emergent data obtained during thematic analysis from interviews that guided MB’s adaptation into MBapp.

## Methods

### Overview

English- or Spanish-speaking women who were between 32 weeks’ gestation and 6 months postpartum were eligible. Consenting participants completed a virtual, individual, semistructured interview exploring their perspectives on perinatal mental health and pregnancy- or childbirth-related education and support [5]. They were also asked to provide feedback on draft versions of MBapp [4]. The interview agenda was not designed to elicit discussion on partners; therefore, partner data were emergent. As has been described previously [4,5], each interview was recorded, transcribed, and, after interrater reliability was confirmed, coded by 2 authors for consensus. Coded data were inputted into NVivo and interpreted using a thematic analytic approach. This paper includes only the emergent themes pertaining to participant-initiated perspectives of MBapp’s utility for their partner or relationship with their partner.

### Ethical Considerations

The study was approved by Women & Infant’s Hospital of Rhode Island’s institutional review board (1737296). Participants signed informed consent forms prior to participation and could opt out at any time. All data collected were deidentified, and participants received a US $50 gift card for completing the qualitative interview.

## Results

As previously reported [4,5], the 25 interviewees were racially and ethnically diverse, with half primiparous, (n=13, 52%), half Spanish-speaking, (n=12, 48%), and half pregnant (n=13, 52%) (Table 1). Of the 25 women, 5 independently expressed, without any explicit prompting, that they saw value in providing MBapp to their partner. In this subgroup, 3 of 5 were Spanish-speaking, 2 of 5 were primiparous, and 3 of 5 were post partum.

Each of these 5 participants mentioned their interest in sharing MBapp with their partner when prompted to suggest ways to enhance the app’s usefulness for perinatal women. The 5 women stated that providing MBapp to a partner would make it more effective as both a perinatal psychoeducational tool and a CBT-based program (Table 2). Some endorsed that MBapp could teach their partners how to improve communication and provide infant care, helping them be a more supportive partner. Others noted that MBapp could help their partners recognize the interplay between relationship dynamics, maternal isolation, and PPD symptoms. Each of these 5 participants was surprised that MBapp was planned to be delivered exclusively to the pregnant person.

**Table 1:** Sociodemographic and obstetric characteristics of study participants (N=25).

Characteristics		Values
**Age (years)**		
	Median (IQR)	29 (24-35)
	Range	20-43
Advanced maternal age (35 years or older at time of delivery), n (%)		6 (24)
**Race, n (%)**		
	Black	5 (20)
	American Indian or Alaska Native	3 (12)
	Asian	2 (8)
	White	6 (24)
	Other^a^	9 (36)
**Ethnicity, n (%)**		
	Hispanic	13 (52)
**Language of interview^b^, n (%)**		
	English	13 (52)
	Spanish	12 (48)
Primiparous (pregnant with first child or recently delivered first child)		13 (52)
**Pregnancy status, n (%)**		
	Pregnant	13 (52)
	Postpartum	12 (48)

^a^Other: multiracial (White and Black): n=4; multiracial (White and American Indian or Alaska Native): n=3; multiracial (Black and American Indian or Alaska Native): n=2.

^b^Four participants spoke a combination of English and Spanish. Their interview language was categorized as the language that most of the interview was conducted in.

**Table 2:** Participant-elicited reasons that MBapp would be useful for their partner.

Themes	Quotes
Improve dyadic communication	“Maybe they’re not knowing [how to] explain to the other partner how they’re acting, like they’re not being assertive enough, or they’re being too aggressive. They can open [the app] up and show them, ‘This is what is going on with us. This is how I feel like you’re treating me,’ so they can get more of a better understanding of how you feel.” (English-speaking postpartum person with multiple children)
Increase partner’s knowledge on infant care	“There is nothing like this for men. How is it possible that we are in the 21st century, and men don’t know how to do things for their baby? My brother is like that. He will say, ‘No, a woman does that.’ And I say, ‘No it’s for men and women.’... There are a lot of men like him, who can’t change a stupid diaper and don’t fulfill their role fifty/fifty. The world could change when things like this [program] are mixed, for men and women.” (Spanish-speaking pregnant person who is a first-time parent)
Effect of perceived partner support and maternal stress and isolation	“The emotional state you’re in after giving birth, well. If you don’t get support from your partner, you get stressed out.... Sometimes the baby starts crying and...many partners don’t support you with that. That’s when you get stressed. There comes a time when the woman starts crying because so many things build up that she explodes, and she feels alone.” (Spanish-speaking pregnant person with a prior child)
Interplay between partner stress and maternal mental health symptoms	“People that are dealing with their spouses, like if they’re stressed with each other, it’s taking a toll on both of the parents, like on their mental health. Like, the mom will be dealing with postpartum [depression].... Fathers [must] understand how you have to do well with them to be doing well with yourself and with your baby” (English-speaking postpartum person with multiple children)

## Discussion

This study presents emergent qualitative data from diverse perinatal women whose feedback was used to optimize MBapp, a novel app-based CBT program. Without prompting, 5 of 25 participants noted that MBapp would be more useful for them if it were also provided to their partners. Had the interview specifically queried about perceptions of partner use and involvement, the proportion of participants who reported MBapp would be more useful as a dyadic than a stand-alone intervention may have been higher. These emergent data align with the well-known bidirectional association between maternal and paternal perinatal mental health symptoms [6,7]. Though a small study has proven the feasibility of delivering in-person MB to both parents simultaneously [8], the feasibility and effectiveness of delivering MBapp—or other USPSTF-recommended psychotherapy [2]—as a digital dyadic intervention to prevent PPD has yet to be explored. Study strengths include a diverse study population and interviews conducted in English or Spanish. Furthermore, the study population can be considered sufficiently sampled per qualitative methodology principles [9]. Study limitations include exclusive recruitment at one obstetric clinic. Expanding data collection can assess whether these findings resonate in other populations. In addition, because the interviews were not intended to discuss partner status—much less partner support—the proportion of participants with partners (and supportive or unsupportive partners) is unknown. Lastly, emergent themes on partners were not identified until the data were analyzed; thus, thematic saturation was not achieved. In summary, this qualitative analysis demonstrates that an evidence-based maternal PPD-prevention intervention may be more useful if delivered simultaneously to both parents, potentially by being an upstream mediator of maternal PPD, as per dyadic coping theory [10]. Our results call for additional research examining not just the feasibility and effectiveness but the mechanisms of effectiveness of digital dyadic or stand-alone partner PPD prevention interventions.

## Abbreviations

CBT: cognitive behavioral therapy
MB: Mothers and Babies
PPD: postpartum depression
USPSTF: United States Preventive Services Task Force
